# Knowledge of mothers regarding children’s vaccinations in Cyprus: A cross-sectional study

**DOI:** 10.1371/journal.pone.0257590

**Published:** 2021-09-20

**Authors:** Maria Kyprianidou, Eleana Tzira, Petros Galanis, Konstantinos Giannakou

**Affiliations:** 1 Department of Health Sciences, School of Sciences, European University Cyprus, Nicosia, Cyprus; 2 Cyprus International Institute for Environmental and Public Health, Cyprus University of Technology, Limassol, Cyprus; 3 Faculty of Nursing, Centre for Health Services Management and Evaluation, National and Kapodistrian University of Athens, Athens, Greece; The Chinese University of Hong Kong, HONG KONG

## Abstract

**Introduction:**

Vaccine hesitancy is identified as one of the top threats to global health. A significant drop of childhood vaccine coverage is reported worldwide. One of the key reasons that influenced mothers’ choice to postpone, or avoid children’s vaccination, is knowledge. This study aimed to assess the level of Cypriot mothers’ knowledge on certain aspects of vaccination of their children, examine the association between vaccination knowledge and selected socio-demographic factors, and lastly assess the association of mothers’ knowledge about vaccination with vaccination coverage and delay, compliance to the recommended schedules, vaccination during pregnancy and mother-pediatrician relationship.

**Methods:**

An online-based cross-sectional study conducted to collect information about socio-demographic characteristics, child’s characteristics, vaccination, and vaccine knowledge, using a self-administered questionnaire. The survey was conducted between April 2020 and June 2020 and the study population included mothers over 18 years old with at least one child (<18 years old) living in Cyprus.

**Results:**

A total of 703 Cypriot mothers participated in the study. Most of the participants stated that they vaccined their children (97%) and the most popular source of information about vaccination was their pediatrician (90%). More than half of the participants (57%) have delayed their child/children vaccination with their pediatrician’s suggestion being the main reason. 36% of mothers had low knowledge while the overall correct rate was 13.6% and the median (IQR) knowledge score was 11 (9–12). Having a medium knowledge about vaccination was associated with having a medium or high income, whilst high knowledge compared to low knowledge was associated with completed a higher education and having a high income. Our analysis showed that the correct knowledge by mothers with regards to vaccination increases the probability of vaccinating their children, following the local recommendations for vaccine dosages, and acquiring and trusting vaccination-related information from their children’s pediatrician.

**Conclusion:**

Our findings show that the majority of mothers in Cyprus had positives perceptions regarding childhood vaccination, as reflected with the high vaccination rate, however, some aspects of mothers’ knowledge of vaccination need to be improved. Public health strategies to promote vaccination, education programs as well as improved communication tools between pediatricians and mothers need to be considered to achieve favorable vaccination attitudes and practices for all mothers in Cyprus.

## Introduction

Vaccination has been regarded as one of the most important public health achievements and one of the most cost-effective interventions for child health promotion that reduces both the morbidity and the mortality from associated vaccine-preventable diseases [1–3]. However, despite ample scientific evidence showing the importance of vaccination, parents still have significant concerns about vaccines and their effects [4, 5]. Parental decisions concerning the vaccination of their children range in various broad categories. Those range from the categorical refusal of any vaccination, intentional delay or selective omission, to full compliance with the entire scheme of routinely recommended vaccinations [6]. Most notably, vaccine hesitancy has risen in recent decades [7–9], leading to a steadily decrease of many levels of childhood vaccine coverage in multiple countries, including the United States, which caused the outbreaks of vaccine-preventable diseases (e.g. measles, pertussis and mumps) [10–17].

Parents are involved in the decision-making process regarding the vaccination of their children; thus, their judgment is crucial. Several studies have examined the knowledge, attitudes, and beliefs of parents of young children with regards to vaccination [18–20]. A common finding of those studies is the lack of knowledge, while the lack of information was considered as a main reason for the parents’ choice to postpone or avoid vaccination [4, 21–23]. Relatively few studies have focused on mothers, a group that is primarily responsible for childcare and vaccine-related decisions [5, 24–26]. Mothers’ knowledge has been proposed as an important factor determining childcare as well as influencing their decisions towards vaccination of children [26–30]. Mothers are often the primary decision makers for healthcare issues of their children, including vaccination [5, 24, 25], and hence our main research question is the knowledge of mothers regarding vaccination.

Cyprus is a small Mediterranean country and the knowledge and information on any issue and especially on health issues is a very important aspect. All the published data are disseminated very quickly in the population of Cyprus, so we believe that this information will benefit the society of Cyprus with a positive response to vaccinations. Having that information and data, the public health authorities will be able to plan the necessary measures, strategies, or interventions regarding the promotion of vaccination in Cyprus. Of interest, a recent large-scale study which was conducted in eighteen European countries concerning vaccine confidence among parents, reported that vaccine confidence was highest in Portugal and Cyprus and lowest in Bulgaria and Poland and specifically 78% of Cypriots were reported as not at all hesitant [31]. However, a recent research study among nurses and midwives in Cyprus identified negative attitudes towards the vaccination of their children with the COVID-19 vaccine [32].

To our knowledge, data on mothers’ knowledge towards the vaccination of their children are not available in Cyprus. Such information is urgently needed, since the reasons for noncompliance with, or non-delivery of vaccinations to eligible children on schedule, and the factors that affect vaccination rates need to be identified. Therefore, to fill this knowledge gap, this study aimed to assess the level of Cypriot mothers’ knowledge on certain aspects of vaccination of their children, examine the association between vaccination knowledge and selected socio-demographic factors, and lastly assess the association of mothers’ knowledge about vaccination with vaccination coverage and delay, compliance to the recommended schedules, vaccination during pregnancy and mother-pediatrician relationship.

## Materials and methods

### Study design and setting

This was an online cross-sectional study. The referent population included mothers over 18 years old with at least one minor (<18 years old) child living in the five government-controlled municipalities of the Republic of Cyprus (Nicosia, Limassol, Larnaca, Paphos and Ammochostos). Mothers who only had adult children were excluded. We focus on mothers’ vaccination knowledge since it is a key factor concerning children healthcare and decisions concerning vaccination. Data collection took place during April 2020-June 2020.

### Sampling

Recruitment occurred using instant messaging apps (e.g., WhatsApp), social media (e.g., Facebook, Instagram), social networking sites (e.g., LinkedIn), and emails, to collect a representative sample from all areas of Cyprus [Nicosia, 52% of the total Cypriot population, Limassol (26%), Larnaca (15%), Paphos (4%), and Ammochostos (3%)]. Thus, a convenience sample was used. This convenience sampling approach was inevitable, due to the quarantine restrictions resulting from the on-going COVID-19 pandemic, which consequently influenced sampling possibilities. Despite the non-probabilistic sampling approach, we have managed to recruit participants from all areas of Cyprus and from different age and socioeconomic strata, thus ensuring a fairly representative sample of the adult female Cypriot population.

### Participants’ characteristics

The information was collected via a self-administered online questionnaire. Data included socio-demographic characteristics (i.e., age, employment status, educational, marital and religion status), characteristics about the children (i.e., number of children, age, and gender), information about vaccination (i.e., following the prescribed doses as indicated by the local recommendations for each vaccine, sources of information about vaccination, trust in the pediatrician, delay of vaccination and reasons for that) and information about vaccine knowledge.

Job status was recorded as private employee, state employee, freelancer and unemployed (i.e., unemployed, housewife, student and retired) while marital status was recorded as never married, married/in cohabitation, or separated/divorced/widowed. Education level was classified in three categories namely, primary education (participants who completed only primary school; <7 years of schooling), secondary education (participants who completed middle or high school; 7–12 years of schooling) and higher education (participants who have a university degree; >12 years of schooling). Salary status was evaluated using the monthly income (based on financial status in Cyprus) and was classified as, no income, low income (≤ €1,500), moderate income (€1,501–2,500); and high income (> €2,500). Religion status was recorded as Christian Orthodox, Christian Catholic, Muslim or other.

The sources of information about vaccination were defined using the question “What is the main source of information for you about vaccinating your children?” with possible answers pediatrician, pharmacist, family doctor, personal doctor, internet and media, and family and friends. The question used to obtain the reasons of possible delay of vaccination was “If you have delayed your child/children vaccination, what were the main reasons?” with possible answers illness, lack of clear information, pediatrician’s suggestion, fear of side effects of the vaccine, increased cost of vaccines, increased cost of medical visit, long distance from the vaccination site or other.

Mothers’ knowledge regarding vaccination was measured by thirteen questions that were formed on the basis of extensive literature search, which evaluated their knowledge about vaccination with three possible answers: “True”, “False”, and “I do not know”. Since vaccination-related knowledge was the most important variable of our study, various types of questions were created assessing mothers’ general knowledge on vaccines. In addition, we included questions relating to controversial subjects that are often related to vaccinations, for example alleged links to autism, harmful chemicals in vaccines, and common misconceptions that are often linked to vaccines (i.e., whether vaccinations can be performed during the summer) (S1 File).

### Ethics approval

The study was approved by the Cyprus National Bioethics Committee (CNBC) (EEBK EΠ 2020.01.82). The application, along with the relevant questionnaire, submitted to the CNBC outlined the study objectives and outcomes, the data collection process and data management, the use of the data, and the expected benefits. All the participants were informed about the aim and objectives of the study before participating at the first page of the questionnaire. The respondents needed to confirm their willingness to participate on a voluntary basis by answering a “Yes” or “No” question on a written informed consent form before being allowed to complete the online self-reporting questionnaire.

### Statistical analysis

Participants’ baseline characteristics are presented as mean ± standard deviation (SD) for continuous variables with normal distributions (i.e., age) and as median (q_1_, q_3_) for continuous measures with skewed distributions (i.e., number of children) while categorical variables (i.e., city of residency, region of residency, marital status, single parent family status, education level, employment, and income status) were presented as absolute (n) and relative (%) frequencies. Normality was tested using Shapiro-Wilk test. Likewise, children’s baseline characteristics are presented as median (q_1_, q_3_) for continuous measures with skewed distributions (i.e., age of children) and as absolute (n) and relative (%) frequencies for categorical variables (i.e., gender of children).

Pearson’s chi square test was employed to detect any differences between the question of vaccine’s knowledge and the categorical baseline characteristics of the participants (i.e., marital status, single parent family, city, region of residency, educational level, and employment and income status). T-test and Kruskal-Wallis rank test were applied to find any differences between the question of vaccine’s knowledge and the normally continuous characteristics of the participants (i.e., age) and not-normally continuous characteristics of the participants (i.e., age of children), respectively.

A knowledge score was created for each participant by scoring the individual knowledge question items, giving a score of 1 for each question correctly answered and 0 for each question answered incorrectly or in case of lack of knowledge (i.e., answered “I do not know”). We calculated the knowledge score of the mothers by adding the points of each of the 13 knowledge items (maximum score 13). We used knowledge score as a continuous variable but also as a categorical variable, by using the quartiles of the knowledge score, as follows: low knowledge (score ≤ 9), moderate knowledge (score 10–11) and good knowledge (score >12).

A bubble graph was used to present the combinations of the correct answers of the 13 questions about the knowledge about vaccination. It is a generalization of the scatter plot, replacing the dots with bubbles, and it is very useful for comparing the associations between data objects in three-numeric-data dimensions, namely, the X-axis data, the Y-axis data, and its size, with larger bubbles indicating larger values.

We performed a multiple logistic regression of maternal vaccination knowledge as a dependent variable after accounting for socio-demographic factors in order to identify the factors which influence the level of vaccination knowledge (base outcome: low knowledge) (Table 3). In additional, a multiple logistic regression analysis was conducted using knowledge level (low, moderate, high) as an independent variable after accounting for different socio-demographic maternal and child factors (i.e., age of mother, age of child, marital status, single parent family status, geographical area, residency, educational, employment and income status) on vaccination coverage and delay, compliance to the recommended schedules, vaccination during pregnancy and mother-pediatrician relationship (Table 4). Finally, we applied a multiple logistic regression analysis using the 13 different knowledge items about vaccination as independent variables after accounting for different socio-demographic maternal and child factors (i.e., age of mother, age of child, marital status, single parent family status, geographical area, residency, educational, employment and income status), on vaccination coverage and delay, compliance to the recommended schedules, vaccination during pregnancy and mother-pediatrician relationship (Table 5).

The statistical significance of the models was assessed using Bonferroni correction. All statistical tests performed were two-sided with the statistical significance level set at α = 0.05. Statistical analysis was conducted using STATA 14.0 (Stata Corp, College Station, TX, USA).

## Results

### Participants’ characteristics

A total of 703 mothers in Cyprus completed the online questionnaire. The socio-demographic characteristics of the respondents are described in Table 1. The median age of the mothers was 35.5 (q_1_ = 32, q_3_ = 39) years old. About 51% of the participants were from the capital of Cyprus, Nicosia, and the majority were residents of urban regions (81%). In addition, 94% of the participants were married, 91% had completed a higher education and about 42% were categorized as having a high monthly average salary. The median number of children was 2 with the largest number being 6. Among the 704 mothers of the study, 8% were single parent family, while a total of 1218 children were reported in the study. The median age of children was 5 years (q_1_ = 2, q_3_ = 9) and 51% were boys (Table 1).

**Table 1:** Socio-demographic characteristics of the mothers and their children (n = 703).

Socio-demographic characteristics	Categories	n (%)
**Median age of mothers, years (IQR) (n = 699)**		35 (32–39)
**Gender of children, n (%)** (**n = 1191)**^a^	Boys	611 (51.3)
	Girls	580 (48.7)
**Median age of children, years (IQR) (n = 1218)**		5 (2–9)
**City of residency, n (%) (n = 700)**	Nicosia	356 (50.9)
	Limassol	184 (26.3)
	Larnaca	106 (15.1)
	Paphos	31 (4.5)
	Ammochostos	23 (3.3)
**Region of residency, n (%) (n = 688)**	Urban	540 (80.8)
	Rural	128 (19.2)
**Marital status of mother, n (%) (n = 703)**	Unmarried	18 (2.6)
	Married / in cohabitation	658 (93.6)
	Divorced / separated / widowed	27 (3.8)
**Single parent family, n (%) (n = 700)**	No	58 (8.3)
	Yes	642 (91.7)
**Education level of mother, n (%) (n = 704)**	Primary	0 (0)
	Secondary	65 (9.2)
	Higher	639 (90.8)
**Employment status of mother, n (%) (n = 698)**	Unemployed	74 (10.6)
	State employee	172 (24.6)
	Private employee	390 (55.9)
	Freelance	62 (8.9)
**Income status of mother, n (%) (n = 703)**	None	23 (3.1)
	Low	163 (23.2)
	Medium	225 (32.1)
	High	292 (41.6)

Abbreviations: SD, standard deviation; IQR, interquartile range.

^a^ Total number of children who were reported by their mothers.

### Vaccination coverage of recommended vaccines

Most of the participants indicated that they vaccinated their children (96.7%) and that they follow the prescribed doses as suggested by the local recommendations for each vaccine (93.3%) (Table 2). The most popular source of information regarding children’s vaccination among the mothers was the pediatrician (89.6%). In addition, more than half of the mothers have delayed for their child/children vaccination (56.9%) with the main reasons of that delay being the pediatrician’s suggestion (32%), the increased costs (32%) or the fear of side effects (10%) (Table 2).

**Table 2:** Mothers’ responses to questions on vaccination status of their children and their own vaccination status during pregnancy, their attitudes to the pediatrician and their information sources about vaccination.

	Categories	n (%)
**Questions on vaccination status and mothers’ vaccination during pregnancy**		
Did you vaccinate your children in the past? (n = 703)	Yes	681 (96.7)
	No	22 (3.3)
Do you faithfully follow the prescribed dosage as indicated by the local recommendations for each vaccine? (n = 703)	Yes	656 (93.3)
	No	47 (6.7)
Have you ever delayed your child/children vaccination? (n = 703)	Yes	419 (56.9)
	No	284 (40.4)
If you have delayed your child/children vaccination, what is the main reason? (n = 361)^b^	Lack of clear information	32 (8.9)
	Pediatrician’s proposal	116 (32.0)
	Fear of vaccine side effects	35 (9.8)
	Increased cost of vaccines / medical examination	115 (31.8)
	Long distance from the vaccination site	5 (1.4)
	Other^a^	8 (2.2)
	Combination of above reasons	50 (13.9)
Have you vaccinated during your pregnancy? (n = 663)	Yes	108 (16.3)
	No	555 (83.7)
**Questions on mothers’ information sources about vaccination**		
Which is your main information source about your children vaccination issues? (n = 700)^c^	Pediatrician	627 (89.6)
	Pharmacist / Family doctor / Personal doctor	21 (3.0)
	Internet and media	31 (4.4)
	Scientific articles	16 (2.3)
	Other^d^	5 (0.7)
**Questions on mothers’ attitudes to the pediatrician**		
I completely trust my child’s pediatrician (n = 664)	Absolutely disagree / disagree	4 (0.6)
	Neither disagree nor agree	40 (6.0)
	Agree / absolutely agree	620 (93.4)
I freely discuss my concerns with the pediatrician (n = 664)	Absolutely disagree / disagree	2 (0.3)
	Neither disagree nor agree	14 (2.1)
	Agree / absolutely agree	648 (97.6)

Abbreviations: SD, standard deviation; IQR, interquartile range.

^a^ Lack of vaccine/vaccination was not allowed due to illness or medication/negligence/workload.

^b^ Total number of reasons who were reported by their mothers.

^c^ Total number of information who were reported by their mothers.

^d^ All above answers/my own knowledge/environment/scientific articles/national vaccination program/European Centre for Disease Prevention and Control (ECDC)/Word Health Organization.

### Socio-economic and demographic characteristics and vaccination knowledge

Knowledge about vaccination-related questions is presented by marital, educational, and single parent family status. Regarding marital status, we only found a statistically significant association for the knowledge item “Systematic vaccination helped reduce or eliminate many infectious diseases worldwide” (p<0.01). Specifically, the largest differences reported among divorced/separated/widowed mothers in which most of them answered correctly (80.8%), about 11.5% noticed that they did not know, and a small percentage answered wrong (7.7%) (S2 File).

We found statistically significant associations among educational and single parent status and the following knowledge items: “Systematic vaccination helped to reduce or eliminate many infectious diseases worldwide”, “Vaccination can be done in summer” and “Vaccination is not needed for diseases that have disappeared”. Apart from this, we reported that the largest percentage of the mothers who answered correctly to the knowledge item “Vaccination can be done when my child has a cold” was those who completed a secondary education (p<0.01) while the majority of the correct answers of the knowledge items “Children would be more resistant if they were not vaccinated” (p = 0.04), “Many vaccines are given too early, leaving the children’s immune system, unable to develop” (p = 0.02) and “Vaccination is not needed for diseases that have disappeared”, were from mothers who completed a higher education (p = 0.01) (S2 File).

Regarding employment and income status, we found statistically significant associations (p<0.05) in most of the knowledge items (S3 File). Specifically, we found that a larger number of mothers (12%) who had a low income answered incorrectly to the knowledge item “Vaccines are unnecessary, as viruses can be treated with antibiotics” compared to mother who had middle (10%) or high income (4%). Regarding the knowledge item “Vaccine for measles/ rubella/ rubella/ mumps (MMR) is associated with autism”, we reported that more than half of the mothers who had a low income did not know to answer if it is true or false (p<0.01).

We found a statistically significant association (p = 0.016) between the age of the mother and the knowledge item “Vaccination can be done when my child has a fever (>38°C)” and a statistically significant association between the age of the children and the knowledge “Vaccination increases the appearance of allergies” (S4 File). Furthermore, we identified statistically significant associations among the 5 geographical areas of Cyprus and five knowledge items, and we reported one statistically significant association among residents of urban and rural regions and the knowledge item “Systematic vaccination helped to reduce or eliminate many infectious diseases worldwide” (S5 File).

Medium knowledge in a mother compared to low knowledge was associated with income. Specifically having a medium knowledge around childhood vaccination was associated with having a medium or high income. Similarly, high knowledge about vaccination compared to low knowledge was associated with completed a higher education and having a high income (Table 3).

**Table 3:** Multiple logistic regression of vaccination knowledge (base outcome: Low knowledge) after accounting for demographics and socio-economic characteristics.

	Medium vs. Low knowledge		High vs. Low knowledge	
Independent variables	Coefficients (95% CI)	p-value	Coefficients (95% CI)	p-value
**Age of mother**	-0.01 (-0.05, 0.02)	0.48	0.02 (-0.02, 0.05)	0.39
**Age of child**	-0.00 (-0.00, 0.00)	0.63	-0.00 (-0.00, 0.00)	0.81
**Marital status**				
*Unmarried (ref)*				
*Married/in cohabitation*	-0.41 (-1.99. 1.18)	0.62	-0.06 (-1.63, 1.50)	0.94
*Divorced/separated/widowed*	0.60 (-1.03, 2.22)	0.47	0.52 (-1.10, 2.14)	0.53
**Single parent family**	-0.49 (-1.75, 0.77)	0.45	0.00 (-1.14, 1.15)	1.00
**Geographical area**				
*Nicosia (ref)*				
*Limassol*	-0.53 (-1.00, -0.05)	**0.03**	-0.48 (-0.93, -0.02)	**0.04**
*Larnaca*	0.68 (-1.26, -0.10)	**0.02**	-0.91 (-1.50, 0.32)	**<0.01**
*Paphos*	0.19 (-0.82, 1.21)	0.71	-0.31 (-1.42, 0.81)	0.59
*Ammochostos*	0.33 (-0.74, 1.40)	0.55	-0.57 (-1.87, 0.73)	0.39
**Residency**				
*Urban (ref)*				
*Rural*	-0.06 (-0.58, 0.46)	0.82	0.05 (-0.46, 0.56)	0.85
**Educational status**				
*Medium education (ref)*				
*High education*	0.20 (-0.51, 0.91)	0.58	0.90 (0.10, 1.72)	**0.03**
**Job status**				
*Unemployed (ref)*				
*State employee*	-0.08 (-0.78, 0.63)	0.83	-0.24 (-0.94, 0.46)	0.51
*Private employee*	-0.00 (-0.81, 0.80)	0.99	-0.09 (-0.88, 0.70)	0.82
*Freelance*	0.09 (-0.85, 1.02)	0.86	-0.11 (-1.04, 0.83)	0.82
**Income status**				
*None/Low income (ref)*				
*Medium income*	0.61 (0.06, 1.17)	**0.03**	0.46 (-0.09, 1.02)	0.10
*High income*	1.14 (0.56, 1.73)	**<0.01**	1.02 (0.45, 1.59)	**<0.01**

Notes: Bold font indicates statistical significance at p<0.05.

### Combinations of the correct answers of the assessment of knowledge about vaccination

**Fig 1:**
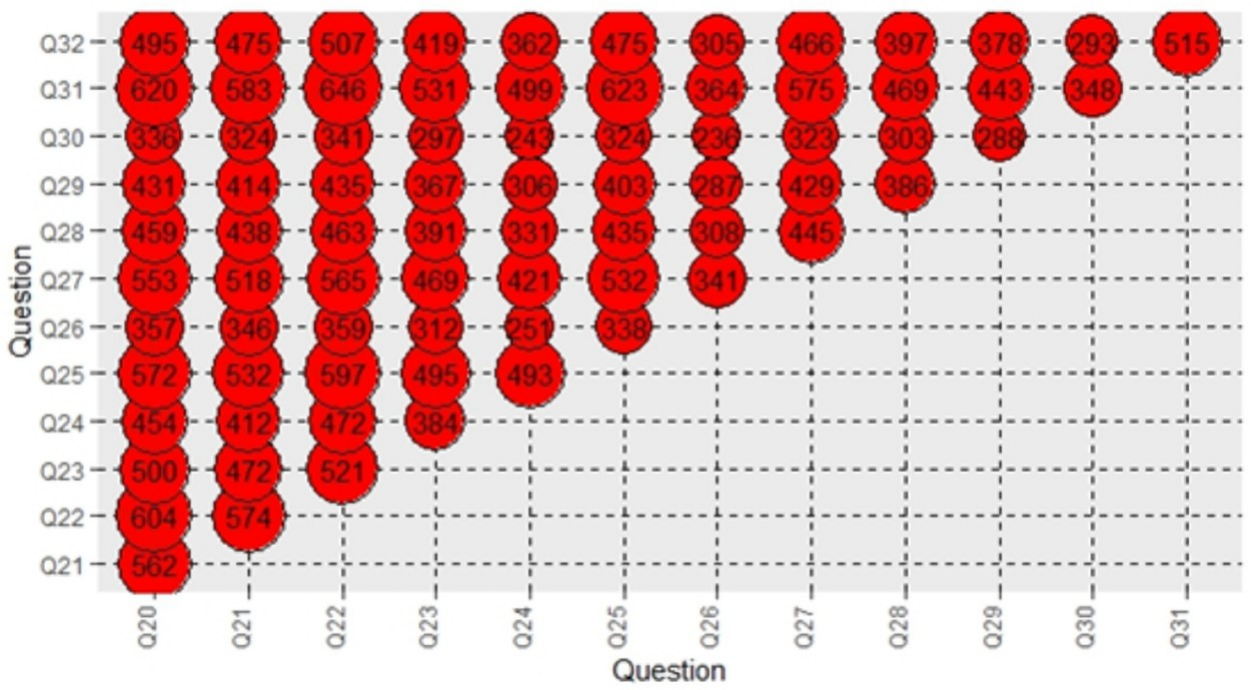
Combinations of the correct answers to the questions about vaccination’s knowledge.

Bubble graph (Fig 1) presents the combinations of the correct answers of the 13 questions about the knowledge about vaccination. The most common correct answers were answers of questions “Systematic vaccination helped to reduce or eliminate many infectious diseases worldwide” and “There is a vaccine to prevent cervical cancer”, followed by questions “Vaccines are unnecessary, as viruses can be treated with antibiotics” and “There is a vaccine to prevent cervical cancer”, and “Vaccines are unnecessary, as viruses can be treated with antibiotics” and “Systematic vaccination helped to reduce or eliminate many infectious diseases worldwide”.

### Knowledge on childhood vaccination

A total of 36% of mothers had low knowledge on childhood vaccination while the overall correct rate was 13.6%. Median (IQR) knowledge score among the total sample of mothers was 11 (9–12).

Table 4 presents the multiple logistic regression models for maternal knowledge of vaccination (moderate and high vs. low) on previous vaccination of their children (Model 1), faithful following of the prescribed dosage as indicated by the local recommendations (Model 2), delay of vaccination (Model 3), vaccination during pregnancy (Model 4), trust of the pediatrician (Model 5), and discussion with the pediatrician (Model 6), after accounting socio-demographic maternal and child factors (i.e., age of the mother, geographical area, residency, marital, job, educational and income status, single parent family status and number of children). We reported that mothers with a moderate knowledge of vaccination have 3.72 (95% CI: 1.08, 12.80) times higher probability of vaccinating their children in the past while mothers with a high knowledge of vaccination have 15.35 (95% CI: 1.94, 121.22) times higher probability, respectively (**Model 1**, Table 4). Similarly, mothers with a high knowledge of vaccination had a higher probability of following the prescribed dosage as indicated by the local recommendations (adjusted OR = 8.92, 95% CI: 2.98, 26.75) (**Model 2**, Table 4), being vaccinated during pregnancy (adjusted OR = 2.97, 95% CI: 1.68, 5.24) (**Model 4**, Table 4). More details are presented in Table 4.

**Table 4:** Multiple logistic regression models [(with odds ratios (ORs) and 95% confidence Intervals (CI)] for the association between mothers’ knowledge about vaccination and vaccination status of children and their mother during pregnancy, mothers’ attitudes to the pediatrician.

Models^a^	Did you vaccinate your children in the past? (model 1)^b^	Do you faithfully follow the prescribed dosage as indicated by the local recommendations for each vaccine? (model 2)^b^	Have you ever delayed your child/children vaccination? (model 3)^b^	Have you vaccinated during your pregnancy? (model 4)^b^	I completely trust my child’s pediatrician. (model 5)^c^	I freely discuss my concerns with the pediatrician. (model 6)^c^
**Level of knowledge**						
Low	*Ref*	*Ref*	*Ref*	*Ref*	*Ref*	*Ref*
Moderate	**3.72 (1.08, 12.80)**	**3.89 (1.69, 9.00)**	1.01 (0.68, 1.53)	1.60 (0.87, 2.92)	**0.26 (0.12, 0.59)**	**0.23 (0.05, 0.97)**
High	**15.35 (1.94, 121.22)**	**8.92 (2.98, 26.75)**	**0.62 (0.42, 0.93)**	**2.97 (1.68, 5.24)**	**0.17 (0.07, 0.44)**	**0.25 (0.06, 1.06)**

^a^ All models adjusted for socio-demographic characteristics (age of mother and children, marital, education, single parent, employment and income status, city, and region of residency).

^b^ yes vs. no.

^c^ agree/absolutely agree vs. neither agree nor disagree/disagree/absolutely disagree.

Notes: Bold font indicates statistical significance at P<0.05 (after Bonferroni correction).

Table 5 displays the multiple logistic regression models which used to model previous vaccination of their children (Model 1), the faithful following of the prescribed dosage as indicated by the local recommendations for each vaccine (Model 2), the delay of vaccination (Model 3), the vaccination during pregnancy (Model 4), the trust to the pediatrician (Model 5), and the discussion with the pediatrician (Model 5) as a linear combination of each question about knowledge of vaccination and the characteristics of the participants (i.e., age of the mother, geographical area, residency, marital, job, educational and income status, single parent family status and number of children). After the evaluation of the statistical significance of the models using Bonferroni correction, the statistically significant associations are presenting in Table 5. Respondents who answered correctly to the questions Q22, Q23, Q25, Q27, Q28 and Q30 are more likely to vaccinate their children compared to those who answered incorrectly (**Model 1**, Table 5). More specifically, people who answered correctly to the question “Systematic vaccination helped to reduce or eliminate many infectious diseases worldwide” had 18.58 times higher risk of vaccinating their children compared to participants who answered incorrectly (95% CI: 5.17, 67.05). When we modeled the faithful following of the prescribed dosage as indicated by the local recommendations for each vaccine as a linear combination of each question about knowledge of vaccination and the characteristics of the study, we reported statistically significant associations for all the questions which mothers answered correctly vs. those who answered incorrectly (**Model 2**, Table 5). Mothers who answered correctly to the questions Q28 (adjusted OR = 0.52, 95% CI: 0.37, 0.75), Q30 (adjusted OR = 0.60, 95% CI: 0.43, 0.83), and Q32 (adjusted OR = 0.53, 95% CI: 0.36, 0.78), had lower risk to not vaccinate their children compared to those who answered incorrectly (**Model 3**, Table 5). Regarding the vaccination during pregnancy (**Model 4**, Table 5), we found that mothers who answered correctly to the question “Vaccines are unnecessary, as viruses can be treated with antibiotics” had 7.34 times higher risk of vaccination during pregnancy compared to those who answered incorrectly. In addition, we reported statistically significant associations in questions Q6 (OR = 2.00, 95% CI: 1.26, 3.16), Q27 (OR = 3.68, 95% CI: 1.54, 8.77), Q28 (OR = 2.77, 95% CI: 1.58, 4.86 and Q29 (OR = 2.25, 95% CI: 1.34, 3.77).

**Table 5:** Multiple logistic regression models [(with odds ratios (ORs) and 95% confidence Intervals (CI)] for the association between mothers’ knowledge about vaccination and vaccination status of children and their mother during pregnancy, mothers’ attitudes to the pediatrician.

Models^a^	Did you vaccinate your children in the past? (model 1)^b^	Do you faithfully follow the prescribed dosage as indicated by the local recommendations for each vaccine? (model 2)^b^	Have you ever delayed your child/children vaccination? (model 3)^b^	Have you vaccinated during your pregnancy? (model 4)^b^	I completely trust my child’s pediatrician. (model 5)^c^	I freely discuss my concerns with the pediatrician. (model 6)^c^
Q20: Vaccines are unnecessary, as viruses can be treated with antibiotics^d^						
OR (95% CI)	1.85 (0.55, 6.18)	**4.30 (2.01, 9.18)**	0.86 (0.50, 1.46)	**7.34 (1.74, 30.95)**	**0.23 (0.11, 0.49)**	0.37 (0.09, 1.50)
Q21: The effectiveness of vaccines has been demonstrated by epidemiological studies^e^						
OR (95% CI)	3.61 (1.35, 9.68)	**5.54 (2.79, 11.01)**	0.59 (0.37, 0.94)	2.88 (1.32, 6.31)	**0.29 (0.15, 0.57)**	0.35 (0.10, 1.18)
Q22: Systematic vaccination helped to reduce or eliminate many infectious diseases worldwide^e^						
OR (95% CI)	**18.58 (5.15, 67.05)**	**11.69 (4.82, 28.38)**	**0.45 (0.21, 0.96)**	4.53 (1.02, 20.10)	**0.18 (0.08, 0.43)**	0.13 (0.03, 0.60)
Q23: Vaccination can be done in summer^e^						
OR (95% CI)	**5.48 (2.06, 14.56)**	**3.33 (1.69, 6.56)**	1.07 (0.72, 1.59)	1.09 (0.63, 1.86)	0.53 (0.27, 1.05)	0.28 (0.09, 0.90)
Q24: Vaccination can be done when my child has a cold^d^						
OR (95% CI)	2.45 (0.92, 6.53)	**0.37 (0.15, 0.94)**	1.59 (1.11, 2.30)	0.69 (0.43, 1.12)	1.34 (0.64, 2.84)	1.39 (0.35, 5.51)
Q25: Vaccination can be done when my child has a fever (>38°C)^d^						
OR (95% CI)	**8.32 (3.04, 22.75)**	**3.01 (1.28, 7.07)**	1.07 (0.62, 1.86)	1.02 (0.50, 2.09)	0.85 (0.33, 2.18)	1
Q26: Vaccine for measles/ rubella/ rubella/ mumps (MMR) is associated with autism^d^						
OR (95% CI)	4.00 (1.35, 11.86)	**4.39 (2.07, 9.29)**	0.72 (0.52, 0.99)	**2.00 (1.26, 3.16)**	0.42 (0.22, 0.81)	0.29 (0.09, 0.97)
Q27: Children would be more resistant if they were not vaccinated^d^						
OR (95% CI)	**6.12 (2.55, 16.66)**	**13.46 (6.63, 27.33)**	0.62 (0.39, 0.97)	**3.68 (1.54, 8.77)**	0.13 (0.07, 0.25)	**0.20 (0.06, 0.66)**
Q28: Many vaccines are given too early, leaving the children’s immune system, unable to develop^d^						
OR (95% CI)	**4.56 (1.68, 12.36)**	**5.40 (2.71, 10.78)**	**0.52 (0.37, 0.75)**	**2.77 (1.58, 4.86)**	**0.15 (0.08, 0.30)**	0.31 (0.10, 0.97)
Q29: The doses of chemicals that are used in the vaccines are dangerous for humans^d^						
OR (95% CI)	2.79 (1.07, 7.29)	**6.71 (3.18, 14.19)**	0.62 (0.44, 0.88)	**2.25 (1.34, 3.77)**	**0.21 (0.11, 0.42)**	0.30 (0.10, 0.97)
Q30: Vaccination increases the appearance of allergies^d^						
OR (95% CI)	**6.24 (1.71, 22.75)**	**6.46 (2.77, 15.07)**	**0.60 (0.43, 0.83)**	1.32 (0.85, 2.04)	**0.29 (0.15, 0.59)**	0.19 (0.05, 0.74)
Q31: There is a vaccine to prevent cervical cancer^e^						
OR (95% CI)	5.01 (0.85, 29.6)	**6.04 (1.51, 24.11)**	1.46 (0.46, 4.59)	2.75 (0.34, 22.08)	**0.31 (0.07, 1.34)**	1
Q32: Vaccination is not needed for diseases that have disappeared^d^						
OR (95% CI)	3.61 (1.40, 9.33)	**3.91 (2.03, 7.53)**	**0.53 (0.36, 0.78)**	1.55 (0.90, 2.67)	**0.35 (0.19, 0.66)**	0.27 (0.09, 0.84)

^a^ All models adjusted for sociodemographic characteristics (age of mother and children, marital, education, single parent, employment and income status, city, and region of residency);

^b^ yes vs. no;

^c^ agree / absolutely agree vs. neither agree nor disagree / disagree / absolutely disagree;

^d^ false vs. true / I don’t know;

^e^ true vs. false / I don’t know;

Bold font indicates statistical significance at P<0.05 (after Bonferroni correction).

Regarding the trust of the pediatrician, we found statistically significant associations with people who answered correctly to questions Q20, Q21, Q22, Q28, Q29, Q30, Q31 and Q32 (**Model 5**, Table 5). Those associations showed a lower risk of the mothers’ trust to the pediatrician for those mothers who answered correctly. More specifically, mothers who answered correctly to the questions “Vaccines are unnecessary, as viruses can be treated with antibiotics”, “The effectiveness of vaccines has been demonstrated by epidemiological studies”, “Systematic vaccination helped to reduce or eliminate many infectious diseases worldwide”, “Many vaccines are given too early, leaving the children’s immune system, unable to develop”, “The doses of chemicals that are used in the vaccines are dangerous for humans”, “Vaccination increases the appearance of allergies”, “There is a vaccine to prevent cervical cancer” and “Vaccination is not needed for diseases that have disappeared” are presented about 77%, 71%, 82%, 85%, 79%, 71%, 69% and 65% lower risk of trusting their pediatrician compared to those who answered incorrectly. In addition, we only found a statistically significant association among mothers who answered correctly in the question “Children would be more resistant if they were not vaccinated” compared to those who answered incorrectly (**Model 6**, Table 5, adjusted OR = 0.20, 95% CI: 0.06, 0.66).

## Discussion

To the best of our knowledge, this is the first study that evaluated the knowledge of mothers in Cyprus concerning the vaccination of their children. Our findings show that most mothers (97%) had positives perceptions regarding childhood vaccination, as reflected with high vaccination rate, and only a small percentage (<3%) of mothers never gave a single vaccination to their children. The most popular source of information about vaccination was pediatrician (90%), while more than half of the participants (57%) have delayed their child/children vaccination based on the pediatrician’s suggestion. A total of 36% of mothers had low knowledge on childhood vaccination and the overall correct rate was 13.6%. Moreover, we found that having a medium knowledge about vaccination was associated with medium or high income while high knowledge about vaccination was associated with completed a higher education and having a high income. Mothers also considered it very important for their children to receive all the recommended vaccines according to the local recommended schedule.

Almost all the participants had positive perceptions about childhood vaccination, and we reported that only 3% of the mothers that participated in the study were vaccine hesitant. This is a very important finding since in many other countries childhood vaccine hesitancy is increasing. Specifically, an epidemiological study which used data from the National Immunization Survey found that 21.8% of parents reported delaying vaccinations for their children [14], while another study reported that parents who were hesitant about childhood vaccination was 25.8% in 2018 and 19.5% in 2019 [33]. Furthermore, another study reported a high prevalence of childhood vaccine hesitancy among Italian parents [10], whilst in a recent study in Peru the corresponding percentage was 9.8% [34].

The median score of knowledge about childhood vaccination of mothers suggests that mothers were generally knowledgeable concerning the importance and benefits of vaccinations and their roles in preventing childhood infectious diseases. Our analysis showed that the correct knowledge of the mothers about vaccination increases the probability of vaccinating their children as well as the constant recommendations of the vaccines dosages as indicated by the local recommendations. Similar findings were found in other studies, highlighting the participants’ knowledge as an essential factor for childhood vaccination [30, 35–39].

Parental educational status has been extensively reported to be an critical determinant of vaccine acceptance and compliance in both developed and developing countries [40]. In the present study a significant association between vaccination status and mothers’ educational level was found. A low education level of mothers was associated with non-adherence to vaccination schedule. In accordance with other studies, the maternal education is a significant predictor of completeness of immunization and important determinant of vaccination coverage [41–43]. Furthermore, a high educational level is associated to a better understanding of vaccine-related information as well as general knowledge of health-related matters and additional education and advice provided directly, or by certain institutions by health care professionals, as well as during general public health campaigns [44]. It is possible therefore for well-educated mothers to have better health literacy and a greater awareness of good healthcare practices. In addition, those recognized the importance of childhood immunization compared to the poorly educated mothers who might have reduced abilities to find, understand, and utilize health-related information.

Our study also revealed a significant difference in mothers’ income status in terms of their knowledge and practices regarding vaccination. Most of the respondents were employed and had access to a regular monthly income, suggesting that their status may contribute to the higher vaccination rate. In line with our results, previous studies have shown that financial barriers are important factors related to vaccination acceptance and utilization whereas higher economic status is linked to a generally better awareness towards immunization, and lower under-vaccination rates [45–50].

Mothers’ knowledge is dependent to a large extent on the expertise of healthcare providers advising upon and administering the vaccines [51], and our data support that statement. Most mothers acquired and trusted vaccination-related information from their children’s pediatrician, highlighting role of physicians to influence maternal decisions and reassuring mothers regarding vaccine safety. Our finding is consistent with the results of several previous studies [5, 7, 52–55]. This emphasizes the doctor’s influence in both giving the correct information and assisting parents to make the right decision whether to have the child vaccinated. Likewise, our results highlight the importance of the trust mothers place in their pediatrician and stresses the vital role they have in effectively communicating issues of immunization, and in disseminating evidence-based vaccine information. Pediatricians have multiple opportunities over many years on clarifying and reaffirming mothers’ correct beliefs about vaccinations and on modifying misconceptions. As we observed, attitudes about childhood vaccination and preferred modes of risk-benefit information can vary depending on several factors such as income and education, hence healthcare providers should be aware about those elements.

Although our study focused on mothers’ knowledge about childhood vaccination, we found that more than half of the mothers (57%) have delayed their child/children vaccination with a significant percentage of them (32%), basing their reason for delay, on the pediatrician’s suggestion. Vaccination delay could cause an alteration in the course of vaccination, leading to non-specific results of vaccines with negative consequences on morbidity and mortality among children [56]. Our findings showed that the main source of vaccination-related information were pediatricians, indicating their important role on mothers’ decisions on childhood vaccination. This finding is consistent with other studies that reported the important role of the physicians in the delivery of vaccinations during childhood, but also their role to influence maternal decisions [5, 7, 24, 25, 53–55, 57]. Pediatricians must keep up to date with the latest developments and disseminate to mothers up to date information on vaccines, in the light of recent scientific studies, and correct mistakes about vaccine hesitancy by using all methods available to them. This is a key finding that must be further considered and evaluated. Our results also emphasize the significance of the trust mothers place in their pediatrician and highlight their key role in communicating information on immunization effectively.

This study has several important implications. Our results suggest that efforts to maintain and improve vaccination rate need to be made by targeting mothers with attitudes and behaviors indicative of vaccine safety concerns, and those with socioeconomic-related risk factors (e.g., educational level and income). Fears about vaccine safety will continue to affect maternal vaccine beliefs and decisions, therefore, an open communication between pediatricians and mothers will serve to improve awareness about vaccine safety and the importance of vaccination. Healthcare providers need to discuss with mothers and convince them about the benefits of vaccines, especially those with high levels of health literacy. To achieve this complex task, healthcare providers need new, more sophisticated communication tools such as social marketing tools and social media to explain the benefits of immunization through vaccination. A collaboration between pediatricians, obstetricians, and midwives to initiate the discussion on childhood vaccination before the birth could be beneficial.

Despite the important research outcomes from this study several limitations should be addressed here. Firstly, this was a cross-sectional study, thus we cannot infer causal relationships between mothers’ knowledge or attitude and the vaccination behavior on their children. Secondly, data were collected using online self-administered questionnaires that limits the sample representativeness and is prone to bias due to misreporting of self-report data. However, the use of a web-based survey is an alternative solution for data collection in periods of social distancing. Thirdly, the data collection was done using a convenience sample through an online tool that limits our study representativeness. Fourthly, all knowledge items included in the study had equal weight in the calculation of vaccination knowledge score.

## Conclusion

In conclusion, the vaccination coverage rate is high in Cyprus, however, some aspects of mothers’ knowledge of vaccination need to be improved. Our findings provide evidence for a better understanding toward mothers’ vaccine hesitancy as knowledge and attitudes of mothers are directly associated with their practice of vaccination. Public health strategies to promote the vaccination, education programs among pediatricians to ensure that they advocate for vaccinations, and improved communication tools between pediatricians and mothers seems to be important components to achieve favorable vaccination attitudes and practices for all mothers in Cyprus.

## Supporting information

S1 File Question about vaccination’s knowledge. (DOCX)

S2 File Mother’s responses to questions about the knowledge of vaccination by marital, educational, and single parent status. (DOCX)

S3 File Mother’s responses to questions about the knowledge of vaccination by employment and income status. (DOCX)

S4 File Mother’s responses to questions about the knowledge of vaccination by age of mother and their children. (DOCX)

S5 File Mother’s responses to questions about the knowledge of vaccination by city and region of residency. (DOCX)
