# Supplementary material for: Knowledge of mothers regarding children’s vaccinations in Cyprus: A cross-sectional study
Source: PLoS One. 2021 Sep 20;16(9):e0257590. doi: 10.1371/journal.pone.0257590 (PMC8452034; doi:10.1371/journal.pone.0257590)
Supplement: S1 File — (DOCX) [file pone.0257590.s001.docx]

| **S1 File.** Question about vaccination’s knowledge. | |
| --- | --- |
| Q20 | Vaccines are unnecessary, as viruses can be treated with antibiotics. |
| Q21 | The effectiveness of vaccines has been demonstrated by epidemiological studies. |
| Q22 | Systematic vaccination helped to reduce or eliminate many infectious diseases worldwide. |
| Q23 | Vaccination can be done in summer. |
| Q24 | Vaccination can be done when my child has a cold. |
| Q25 | Vaccination can be done when my child has a fever (>38°C). |
| Q26 | Vaccine for measles/ rubella/ rubella/ mumps (MMR) is associated with autism. |
| Q27 | Children would be more resistant if they were not vaccinated. |
| Q28 | Many vaccines are given too early, leaving the children's immune system, unable to develop. |
| Q29 | The doses of chemicals that are used in the vaccines are dangerous for humans. |
| Q30 | Vaccination increases the appearance of allergies. |
| Q31 | There is a vaccine to prevent cervical cancer. |
| Q32 | Vaccination is not needed for diseases that have disappeared. |
