# Supplementary material for: Knowledge of mothers regarding children’s vaccinations in Cyprus: A cross-sectional study
Source: PLoS One. 2021 Sep 20;16(9):e0257590. doi: 10.1371/journal.pone.0257590 (PMC8452034; doi:10.1371/journal.pone.0257590)
Supplement: S2 File — (DOCX) [file pone.0257590.s002.docx]

| **S2 File.** Mother’s responses to questions about the knowledge of vaccination by marital, educational, and single parent status. | | | | | | | | | | | | | |
| --- | --- | --- | --- | --- | --- | --- | --- | --- | --- | --- | --- | --- | --- |
|  | **Marital status** | | | | | **Educational status** | | | | | **Single parent status** | | |
|  | Total | Unmarried | Married/In cohabitation | Divorced/Separated/Widowed | p-value | Total | Secondary | Higher | p-value | Total | No | Yes | p-value |
| **Vaccines are unnecessary, as viruses can be treated with antibiotics.** | | | | | | | | | | | | | |
| T | 18 (2.6) | 0 (0.0) | 18 (2.7) | 0 (0.0) | 0.76 | 18 (2.6) | 2 (3.1) | 16 (2.5) | 0.15 | 18 (2.6) | 15 (2.3) | 3 (5.2) | 0.30 |
| F | 630 (89.6) | 16 (88.9) | 509 (89.7) | 24 (88.9) |  | 631 (89.6) | 54 (83.1) | 577 (90.3) |  | 628 (89.7) | 579 (90.2) | 49 (84.5) |  |
| I | 55 (7.8) | 2 (11.1) | 50 (7.6) | 3 (11.1) |  | 55 (7.8) | 9 (13.9) | 46 (7.2) |  | 54 (7.7) | 48 7.5) | 6 (10.3) |  |
| **The effectiveness of vaccines has been demonstrated by epidemiological studies.** | | | | | | | | | | | | | |
| T | 587 (83.6) | 15 (83.3) | 551 (83.9) | 21 (77.8) | 0.86 | 588 (83.6) | 49 (75.4) | 539 (84.5) | 0.07 | 585 (83.7) | 15 (2.3) | 3 (5.2) | 0.10 |
| F | 18 (2.6) | 0 (0.0) | 17 (2.6) | 1 (3.7) |  | 18 (2.6) | 1 (1.5) | 17 (2.7) |  | 18 (2.6) | 579 (90.2) | 49 (84.5) |  |
| I | 97 (13.8) | 3 (16.7) | 89 (13.6) | 5 (18.5) |  | 97 (13.8) | 15 (23.1) | 82 (12.9) |  | 96 (13.7) | 48 (7.5) | 6 (10.3) |  |
| **Systematic vaccination helped to reduce or eliminate many infectious diseases worldwide.** | | | | | | | | | | | | | |
| T | 656 (93.5) | 15 (83.3) | 620 (94.2) | 21 (80.8) | **<0.01** | 657 (93.5) | 54 (83.1) | 603 (94.5) | **<0.01** | 654 (93.6) | 608 (94.7) | 46 (80.7) | **<0.01** |
| F | 22 (3.1) | 3 (16.7) | 17 (2.6) | 2 (7.7) |  | 22 (3.1) | 6 (9.2) | 16 (2.5) |  | 22 (3.1) | 17 (2.7) | 5 (8.8) |  |
| I | 24 (3.4) | 0 (0.0) | 21 (3.2) | 3 (11.5) |  | 24 (3.4) | 5 (7.7) | 19 (3.0) |  | 23 (3.3) | 17 (2.7) | 6 (10.5) |  |
| **Vaccination can be done in summer.** | | | | | | | | | | | | | |
| T | 537 (76.5) | 15 (83.3) | 507 (77.2) | 17 (63.0) | 0.48 | 538 (76.5) | 33 (50.8) | 505 (79.2) | **<0.01** | 536 (76.7) | 501 (78.2) | 35 (60.3) | **<0.01** |
| F | 20 (2.9) | 3 (16.7) | 18 (2.7) | 1 (3.7) |  | 20 (2.8) | 5 (7.7) | 15 (2.4) |  | 20 (2.9) | 18 (2.8) | 2 (3.5) |  |
| I | 145 (20.7) | 0 (0.0) | 132 (20.1) | 9 (33.3) |  | 145 (20.6) | 27 (41.5) | 118 (18.5) |  | 143 (20.5) | 122 (19.0) | 21 (36.2) |  |
| **Vaccination can be done when my child has a cold.** | | | | | | | | | | | | | |
| T | 113 (16.1) | 0 (0.0) | 110 (16.7) | 3 (11.1) | 0.22 | 113 (16.1) | 5 (7.7) | 108 (16.9) | **<0.01** | 113 (16.1) | 109 (17.0) | 4 (6.9) | 0.09 |
| F | 513 (73.0) | 17 (94.4) | 474 (72.0) | 22 (81.5) |  | 514 (73.0) | 59 (90.8) | 455 (71.2) |  | 510 (72.9) | 461 (71.8) | 49 (84.5) |  |
| I | 77 (11.0) | 1 (5.6) | 74 (11.3) | 2 (7.4) |  | 77 (10.9) | 1 (1.5) | 76 (11.9) |  | 77 (11.0) | 72 (11.2) | 5 (8.6) |  |
| **Vaccination can be done when my child has a fever (>38°C).** | | | | | | | | | | | | | |
| T | 19 (2.7) | 1 (5.6) | 18 (2.7) | 0 (0.0) | 0.63 | 19 (2.7) | 2 (3.1) | 17 (2.7) | 0.45 | 19 (2.7) | 19 (3.0) | 0 (0.0) | 0.41 |
| F | 635 (90.5) | 17 (94.4) | 593 (90.3) | 25 (92.6) |  | 636 (90.5) | 61 (93.9) | 575 (90.1) |  | 632 (90.4) | 578 (90.2) | 54 (93.1) |  |
| I | 48 (6.8) | 0 (0.0) | 46 (7.0) | 2 (7.4) |  | 48 (6.8) | 2 (3.1) | 46 (7.2) |  | 48 (6.9) | 44 (6.9) | 4 (6.9) |  |
| **Vaccine for measles/ rubella/ rubella/ mumps (MMR) is associated with autism.** | | | | | | | | | | | | | |
| T | 39 (5.6) | 1 (5.6) | 36 (5.5) | 2 (7.4) | 0.85 | 39 (5.6) | 4 (6.2) | 35 (5.5) | 0.10 | 39 (5.6) | 34 (5.3) | 5 (8.6) | 0.57 |
| F | 367 (52.4) | 8 (44.4) | 343 (52.3) | 16 (59.3) |  | 368 (52.4) | 26 (40.0) | 342 (53.7) |  | 367 (52.6) | 337 (52.7) | 30 (51.7) |  |
| I | 295 (42.1) | 9 (50.0) | 277 (42.2) | 9 (33.3) |  | 295 (42.0) | 35 (53.9) | 260 (40.8) |  | 292 (41.8) | 269 (42.0) | 23 (40.0) |  |
| **Children would be more resistant if they were not vaccinated.** | | | | | | | | | | | | | |
| T | 37 (5.3) | 2 (11.1) | 32 (4.9) | 3 (11.1) | 0.42 | 37 (5.3) | 5 (7.8) | 32 (5.0) | **0.04** | 37 (5.3) | 31 (4.8) | 6 (10.3) | 0.19 |
| F | 583 (83.1) | 13 (72.2) | 549 (83.6) | 21 (77.8) |  | 584 (83.1) | 46 (71.9) | 538 (84.2) |  | 580 (83.0) | 535 (83.5) | 45 (77.6) |  |
| I | 82 (11.7) | 3 (16.7) | 76 (11.6) | 3 (11.1) |  | 82 (11.7) | 13 (20.3) | 69 (10.8) |  | 82 (11.7) | 75 (11.7) | 7 (12.1) |  |
| **Many vaccines are given too early, leaving the children's immune system, unable to develop.** | | | | | | | | | | | | | |
| T | 45 (6.4) | 3 (16.7) | 40 (6.1) | 2 (7.4) | 0.34 | 45 (6.4) | 4 (6.2) | 41 (6.4) | **0.02** | 45 (6.4) | 38 (5.9) | 7 (12.1) | 0.18 |
| F | 476 (67.8) | 9 (50.0) | 448 (68.2) | 19 (70.4) |  | 477 (67.9) | 35 (53.9) | 442 (69.3) |  | 474 (67.8) | 436 (68.0) | 38 (65.5) |  |
| I | 181 (25.8) | 6 (33.3) | 169 (25.7) | 6 (22.2) |  | 181 (25.8) | 26 (40.0) | 155 (24.3) |  | 180 (25.8) | 167 (26.1) | 13 (22.4) |  |
| **The doses of chemicals that are used in the vaccines are dangerous for humans.** | | | | | | | | | | | | | |
| T | 41 (5.8) | 3 (16.7) | 40 (6.1) | 2 (7.4) | 0.91 | 41 (5.8) | 5 (7.7) | 36 (5.6) | 0.08 | 41 (5.9) | 35 (5.5) | 6 (10.3) | 0.27 |
| F | 446 (63.5) | 9 (50.0) | 448 (68.2) | 19 (70.4) |  | 447 (63.6) | 33 (50.8) | 414 (64.9) |  | 445 (63.7) | 408 (63.7) | 37 (63.8) |  |
| I | 215 (30.6) | 6 (33.3) | 169 (25.7) | 6 (22.2) |  | 215 (30.6) | 27 (41.5) | 188 (29.5) |  | 213 (30.5) | 198 (30.9) | 15 (25.9) |  |
| **Vaccination increases the appearance of allergies.** | | | | | | | | | | | | | |
| T | 41 (5.8) | 3 (16.7) | 35 (5.3) | 3 (11.1) | 0.08 | 41 (5.8) | 8 (12.3) | 33 (5.2) | 0.06 | 41 (5.9) | 34 (5.3) | 7 (12.1) | 0.08 |
| F | 351 (50.0) | 8 (44.4) | 326 (49.6) | 17 (63.0) |  | 351 (49.9) | 31 (47.7) | 320 (50.2) |  | 350 (50.1) | 320 (49.9) | 30 (51.7) |  |
| I | 310 (44.2) | 7 (38.9) | 296 (45.1) | 7 (25.9) |  | 311 (44.2) | 26 (40.0) | 285 (44.7) |  | 308 (44.1) | 287 (44.8) | 21 (36.2) |  |
| **There is a vaccine to prevent cervical cancer.** | | | | | | | | | | | | | |
| T | 685 (97.6) | 17 (94.4) | 642 (97.7) | 26 (96.3) | 0.76 | 686 (97.6) | 61 (95.3) | 625 (97.8) | 0.23 | 683 (97.7) | 629 (98.1) | 54 (93.1) | **0.01** |
| F | 3 (0.4) | 0 (0.0) | 3 (0.5) | 0 (0.0) |  | 3 (0.4) | 0 (0.0) | 3 (0.5) |  | 3 (0.4) | 3 (0.5) | 0 (0.0) |  |
| I | 14 (2.0) | 1 (5.6) | 12 (1.8) | 1 (3.7) |  | 14 (2.0) | 3 (4.7) | 11 (1.7) |  | 13 (1.9) | 9 (1.4) | 4 (6.9) |  |
| **Vaccination is not needed for diseases that have disappeared** | | | | | | | | | | | | | |
| T | 53 (7.5) | 3 (16.7) | 47 (7.1) | 3 (11.1) | 0.47 | 53 (7.5) | 8 (12.3) | 45 (7.0) | **0.01** | 53 (7.8) | 45 (7.0) | 8 (13.8) | **0.02** |
| F | 520 (74.0) | 13 (72.2) | 489 (74.3) | 18 (66.7) |  | 521 (74.0) | 38 (58.5) | 483 (75.6) |  | 519 (74.1) | 485 (75.6) | 34 (58.6) |  |
| I | 130 (18.5) | 2 (11.1) | 122 (18.5) | 6 (22.2) |  | 130 (18.5) | 19 (29.2) | 111 (17.4) |  | 128 (18.3) | 112 (17.5) | 16 (27.6) |  |
| Abbreviations: T, true; F, false; I, I don’t know; Bold font indicates statistical significance (p<0.05). | | | | | | | | | | | | | |
