# Supplementary material for: Knowledge of mothers regarding children’s vaccinations in Cyprus: A cross-sectional study
Source: PLoS One. 2021 Sep 20;16(9):e0257590. doi: 10.1371/journal.pone.0257590 (PMC8452034; doi:10.1371/journal.pone.0257590)
Supplement: S3 File — (DOCX) [file pone.0257590.s003.docx]

| **S3 File.** Mother’s responses to questions about the knowledge of vaccination by employment and income status. | | | | | | | | | | | | |
| --- | --- | --- | --- | --- | --- | --- | --- | --- | --- | --- | --- | --- |
|  | **Employment status** | | | | | | **Income status** | | | | | |
|  | Total | Private employee | State employee | Freelance | Unemployed | p-value | Total | None | Low | Middle | High | p-value |
| **Vaccines are unnecessary, as viruses can be treated with antibiotics.** | | | | | | | | | | | | |
| T | 18 (2.6) | 9 (2.3) | 2 (1.2) | 2 (3.2) | 5 (6.8) | 0.18 | 18 (2.6) | 2 (9.1) | 5 (3.1) | 4 (1.8) | 7 (2.4) | **<0.01** |
| F | 625 (89.5) | 349 (89.5) | 156 (90.7) | 58 (93.6) | 62 (83.8) |  | 629 (89.6) | 19 (86.4) | 138 (84.7) | 198 (88.0) | 274 (93.8) |  |
| I | 55 (7.9) | 32 (7.9) | 14 (8.1) | 2 (3.2) | 7 (9.5) |  | 55 (7.8) | 1 (4.6) | 20 (12.3) | 23 (10.2) | 11 (3.8) |  |
| **The effectiveness of vaccines has been demonstrated by epidemiological studies.** | | | | | | | | | | | | |
| T | 583 (83.6) | 323 (82.8) | 155 (90.6) | 52 (83.9) | 53 (71.6) | **<0.01** | 586 (83.6) | 17 (77.3) | 122 (74.9) | 185 (82.2) | 262 (90) | **<0.01** |
| F | 18 (2.6) | 1 (1.8) | 1 (0.6) | 3 (4.8) | 7 (9.5) |  | 18 (2.6) | 1 (4.6) | 6 (3.7) | 7 (3.1) | 4 (1.4) |  |
| I | 96 (13.8) | 60 (15.4) | 15 (8.8) | 7 (11.3) | 14 (8.9) |  | 97 (13.8) | 4 (18.2) | 35 (21.5) | 33 (14.7) | 25 (8.6) |  |
| **Systematic vaccination helped to reduce or eliminate many infectious diseases worldwide.** | | | | | | | | | | | | |
| T | 652 (93.5) | 370 (94.9) | 165 (96.5) | 56 (90.3) | 61 (82.4) | **<0.01** | 655 (93.4) | 20 (90.9) | 138 (84.7) | 212 (94.6) | 285 (97.6) | **<0.01** |
| F | 22 (3.2) | 9 (2.3) | 2 (1.2) | 4 (4.8) | 8 (10.8) |  | 22 (3.1) | 1 (4.6) | 11 (6.8) | 6 (2.7) | 4 (1.4) |  |
| I | 23 (3.8) | 11 (2.8) | 4 (2.3) | 3 (4.8) | 5 (6.8) |  | 24 (3.5) | 1 (4.6) | 14 (8.6) | 6 (2.7) | 3 (1.0) |  |
| **Vaccination can be done in summer.** | | | | | | | | | | | | |
| T | 534 (76.6) | 302 (77.6) | 134 (77.9) | 48 (77.4) | 50 (67.6) | **0.02** | 537 (76.6) | 16 (72.7) | 105 (64.4) | 174 (77.7) | 242 (82.9) | **<0.01** |
| F | 20 (2.9) | 10 (2.6) | 3 (1.7) | 0 (0.0) | 7 (9.5) |  | 20 (2.9) | 3 (13.6) | 5 (3.1) | 5 (2.2) | 7 (2.4) |  |
| I | 143 (20.5) | 77 (19.8) | 35 (20.4) | 14 (22.6) | 17 (23) |  | 144 (20.5) | 3 (13.6) | 53 (32.5) | 45 (20.1) | 43 (14.7) |  |
| **Vaccination can be done when my child has a cold.** | | | | | | | | | | | | |
| T | 112 (16.1) | 61 (15.6) | 34 (19.8) | 13 (21.0) | 4 (5.4) | **<0.01** | 113 (16.1) | 2 (9.1) | 13 (8.0) | 37 (16.4) | 61 (20.9) | **<0.01** |
| F | 509 (72.9) | 286 (73.3) | 113 (65.7) | 41 (66.1) | 69 (93.2) |  | 512 (72.9) | 18 (81.8) | 139 (85.3) | 168 (74.7) | 187 (64) |  |
| I | 77 (11.0) | 43 (11.0) | 25 (14.5) | 8 (12.9) | 1 (1.4) |  | 77 (11.0) | 2 (9.1) | 11 (6.8) | 20 (8.9) | 44 (15.1) |  |
| **Vaccination can be done when my child has a fever (>38°C).** | | | | | | | | | | | | |
| T | 19 (2.7) | 10 (2.6) | 4 (2.3) | 3 (4.8) | 2 (2.7) | 0.34 | 19 (2.7) | 1 (4.6) | 4 (2.5) | 8 (3.6) | 6 (2.1) | 0.89 |
| F | 630 (90.4) | 351 (90.2) | 156 (90.7) | 52 (83.9) | 71 (96) |  | 634 (90.4) | 20 (90.9) | 145 (89.5) | 204 (90.7) | 265 (90.8) |  |
| I | 48 (6.9) | 28 (7.2) | 12 (7.0) | 7 (11.3) | 1 (1.4) |  | 48 (6.9) | 1 (4.6) | 13 (8.0) | 13 (5.8) | 21 (7.2) |  |
| **Vaccine for measles/ rubella/ rubella/ mumps (MMR) is associated with autism.** | | | | | | | | | | | | |
| T | 39 (5.5) | 17 (4.4) | 11 (6.4) | 5 (8.1) | 6(8.1) | 0.36 | 39 (5.6) | 2 (9.1) | 13 (8.8) | 14 (6.3) | 10 (3.4) | **<0.01** |
| F | 366 (52.6) | 203 (52.3) | 93 (54.1) | 37 (59.7) | 33 (44.6) |  | 367 (52.4) | 10 (45.5) | 68 (41.7) | 108 (48.2) | 181 (62.2) |  |
| I | 291 (41.8) | 168 (43.3) | 68 (39.5) | 20 (32.3) | 35 (47.3) |  | 294 (42.0) | 10 (45.5) | 82 (50.3) | 102 (45.5) | 100 (34.4) |  |
| **Children would be more resistant if they were not vaccinated.** | | | | | | | | | | | | |
| T | 37 (5.3) | 16 (4.1) | 6 (3.5) | 5 (8.1) | 10 (13.5) | **0.04** | 37 (5.3) | 2 (9.1) | 11 (6.8) | 13 (5.8) | 11 (3.8) | **0.03** |
| F | 580 (83.2) | 329 (84.6) | 146 (84.9) | 50 (80.7) | 55 (74.3) |  | 583 (83.2) | 18 (81.8) | 128 (78.5) | 177 (79.0) | 260 (89) |  |
| I | 80 (11.5) | 40 (11.3) | 20 (11.6) | 7 (11.3) | 9 (12.2) |  | 81 (11.5) | 2 (9.7) | 24 (14.7) | 34 (15.2) | 21 (7.2) |  |
| **Many vaccines are given too early, leaving the children's immune system, unable to develop.** | | | | | | | | | | | | |
| T | 45 (6.5) | 18 (4.6) | 8 (4.7) | 6 (9.7) | 13 (17.6) | **<0.01** | 45 (6.4) | 5 (22.7) | 14 (8.6) | 14 (6.3) | 12 (4.1) | **<0.01** |
| F | 472 (67.7) | 261 (67.1) | 119 (69.2) | 46 (74.2) | 46 (62.2) |  | 476 (67.9) | 10 (45.5) | 102 (62.6) | 148 (66.1) | 216 (74) |  |
| I | 180 (25.8) | 110 (28.3) | 45 (26.2) | 10 (16.1) | 15 (20.3) |  | 180 (25.7) | 7 (31.8) | 47 (28.8) | 62 (27.7) | 64 (21.9) |  |
| **The doses of chemicals that are used in the vaccines are dangerous for humans.** | | | | | | | | | | | | |
| T | 41 (5.9) | 19 (4.9) | 5 (2.9) | 6 (9.7) | 11 (14.9) | **<0.01** | 41 (5.9) | 3 (13.6) | 18 (11.1) | 12 (5.3) | 8 (2.7) | **<0.01** |
| F | 443 (63.6) | 254 (65.3) | 118 (68.6) | 38 (61.3) | 33 (44.6) |  | 446 (63.9) | 10 (45.5) | 82 (50.6) | 135 (60.0) | 219 (75) |  |
| I | 213 (30.6) | 116 (29.8) | 49 (28.5) | 18 (29.0) | 30 (40.5) |  | 211 (30.2) | 9 (40.9) | 62 (38.3) | 78 (34.7) | 65 (22.3) |  |
| **Vaccination increases the appearance of allergies.** | | | | | | | | | | | | |
| T | 41 (5.9) | 16 (4.1) | 8 (4.7) | 5 (8.1) | 12 (16.2) | **<0.01** | 41 (5.8) | 5 (22.7) | 13 (8.0) | 14 (6.3) | 9 (3.1) | **<0.01** |
| F | 348 (49.9) | 188 (48.3) | 95 (55.2) | 33 (53.2) | 32 (43.2) |  | 350 (49.9) | 7 (31.8) | 68 (41.7) | 115 (51.3) | 160 (54.8) |  |
| I | 308 (44.2) | 185 (47.6) | 69 (40.1) | 24 (38.7) | 30 (40.5) |  | 310 (44.3) | 10 (45.5) | 82 (50.3) | 95 (42.4) | 123 (42.1) |  |
| **There is a vaccine to prevent cervical cancer.** | | | | | | | | | | | | |
| T | 680 (97.6) | 380 (97.4) | 171 (99.4) | 59 (95.2) | 70 (95.9) | 0.30 | 684 (95.6) | 20 (95.2) | 153 (93.9) | 222 (98.7) | 289 (99) | **<0.01** |
| F | 3 (0.4) | 1 (0.3) | 0 (0.0) | 1 (1.6) | 1 (1.4) |  | 3 (0.4) | 0 (0.0) | 1 (0.6) | 2 (0.9) | 0 (0.0) |  |
| I | 14 (2.0) | 9 (2.3) | 1 (0.6) | 2 (3.2) | 2 (2.7) |  | 14 (4.0) | 1 (4.8) | 9 (5.5) | 1 (0.4) | 3 (1.0) |  |
| **Vaccination is not needed for diseases that have disappeared** | | | | | | | | | | | | |
| T | 53 (7.6) | 27 (6.9) | 11 (6.4) | 6 (9.7) | 9 (12.2) | 0.52 | 53 (11.8) | 2 (9.1) | 20 (12.3) | 16 (7.1) | 15 (5.1) | 0.11 |
| F | 518 (74.2) | 288 (73.9) | 132 (76.7) | 48 (77.4) | 50 (67.6) |  | 519 (73.3) | 15 (68.2) | 110 (67.5) | 165 (73.3) | 229 (78.4) |  |
| I | 127 (18.2) | 75 (19.2) | 29 (16.9) | 8 (12.9) | 15 (20.3) |  | 130 (14.9) | 5 (22.7) | 33 (20.3) | 44 (19.6) | 48 (16.4) |  |
| Abbreviations: T, true; F, false; I, I don’t know; Bold font indicates statistical significance (p<0.05). | | | | | | | | | | | | |
