# Supplementary material for: Knowledge of mothers regarding children’s vaccinations in Cyprus: A cross-sectional study
Source: PLoS One. 2021 Sep 20;16(9):e0257590. doi: 10.1371/journal.pone.0257590 (PMC8452034; doi:10.1371/journal.pone.0257590)
Supplement: S4 File — (DOCX) [file pone.0257590.s004.docx]

| **S4 File.** Mother’s responses to questions about the knowledge of vaccination by age of mother and their children. | | | | |
| --- | --- | --- | --- | --- |
|  | **Age of mother (IQR)** | **p-value**^a^ | **Age of children (months) (IQR)** | **p-valu**e^b^ |
| **Vaccines are unnecessary, as viruses can be treated with antibiotics.** | | | | |
| **T** | 34.5 (31, 36) | 0.36 | 30 (12, 132) | 0.14 |
| **F** | 35 (32, 39) |  | 60 (24, 108) |  |
| **I** | 37 (32, 41) |  | 72 (36, 108) |  |
| **The effectiveness of vaccines has been demonstrated by epidemiological studies.** | | | | |
| **T** | 35 (32, 39) | 0.41 | 60 (24, 108) | 0.12 |
| **F** | 35 (31, 39) |  | 36 (12, 72) |  |
| **I** | 34 (31, 40) |  | 72 (36, 120) |  |
| **Systematic vaccination helped to reduce or eliminate many infectious diseases worldwide.** | | | | |
| **T** | 35 (32, 39) | 0.48 | 60 (24, 108) | 0.51 |
| **F** | 34.5 (31, 40) |  | 36 (24, 96) |  |
| **I** | 34 (29, 39.5) |  | 60 (36, 96) |  |
| **Vaccination can be done in summer.** | | |  | |
| **T** | 35 (32, 39) | 0.43 | 60 (24, 108) | 0.38 |
| **F** | 38 (31, 40) |  | 36 (30, 132) |  |
| **I** | 35 (31, 39) |  | 48 (24, 108) |  |
| **Vaccination can be done when my child has a cold.** | | | | |
| **T** | 35 (32, 39) | 0.44 | 60 (36, 96) | 0.85 |
| **F** | 35 (32, 40) |  | 60 (24, 108) |  |
| **I** | 34 (31, 39) |  | 72 (36, 120) |  |
| **Vaccination can be done when my child has a fever (>38°C).** | | | | |
| **T** | 36 (32, 39) | **0.02** | 48 (24, 132) | 0.81 |
| **F** | 35 (32, 39) |  | 60 (24, 108) |  |
| **I** | 33 (29, 35) |  | 57 (27, 120) |  |
| **Vaccine for measles/ rubella/ rubella/ mumps (MMR) is associated with autism.** | | | | |
| **T** | 34 (32, 40) | 0.14 | 48 (24, 132) | 0.56 |
| **F** | 36 (32, 39) |  | 72 (30, 108) |  |
| **I** | 35 (31, 39) |  | 60 (24, 108) |  |
| **Children would be more resistant if they were not vaccinated.** | | | | |
| **T** | 34 (31, 39) | 0.62 | 48 (17, 108) | 0.52 |
| **F** | 35 (32, 39) |  | 60 (27, 108) |  |
| **I** | 36 (32, 40) |  | 60 (24, 108) |  |
| **Many vaccines are given too early, leaving the children's immune system, unable to develop.** | | | | |
| **T** | 35 (31, 39) | 0.85 | 48 (24, 108) | 0.44 |
| **F** | 35 (32, 39) |  | 60 (30, 120) |  |
| **I** | 35 (32, 40) |  | 60 (24, 108) |  |
| **The doses of chemicals that are used in the vaccines are dangerous for humans.** | | | | |
| **T** | 36 (31, 40) | 0.50 | 72 (24, 132) | 0.87 |
| **F** | 35 (32, 39) |  | 60 (24, 108) |  |
| **I** | 35 (31, 39) |  | 60 (24, 108) |  |
| **Vaccination increases the appearance of allergies.** | | | | |
| **T** | 35 (30, 40) |  | 60 (24, 108) |  |
| **F** | 35 (32, 39) |  | 72 (36, 120) |  |
| **I** | 35 (32, 39) |  | 48 (24, 96) |  |
| **There is a vaccine to prevent cervical cancer.** | | | | |
| **T** | 35 (32, 39) | 0.17 | 60 (24, 108) | 0.82 |
| **F** | 33 (28, 35) |  | 72 (36, 204) |  |
| **I** | 33 (29, 38) |  | 24 (18, 51) |  |
| **Vaccination is not needed for diseases that have disappeared.** | | | | |
| **T** | 35 (31, 40) | 0.82 | 72 (24, 144) | 0.63 |
| **F** | 35 (32, 39) |  | 60 (30, 108) |  |
| **I** | 35 (31, 40) |  | 48 (24, 108) |  |
| Abbreviations: T, true; F, false; I, I don’t know; SD, standard deviation; IQR, interquartile range; ^a^ One-way analysis of variance (ANOVA); ^b^ Kruskal–Wallis rank test; Bold font indicates statistical significance (p<0.05). | | | | |
