# Supplementary material for: Knowledge of mothers regarding children’s vaccinations in Cyprus: A cross-sectional study
Source: PLoS One. 2021 Sep 20;16(9):e0257590. doi: 10.1371/journal.pone.0257590 (PMC8452034; doi:10.1371/journal.pone.0257590)
Supplement: S5 File — (DOCX) [file pone.0257590.s005.docx]

| **S5 File.** Mother’s responses to questions about the knowledge of vaccination by city and region of residency. | | | | | | | | | | | |
| --- | --- | --- | --- | --- | --- | --- | --- | --- | --- | --- | --- |
|  | **City of residency** | | | | | |  | **Region of residency** | | | |
|  | **Total** | **Nicosia** | **Limassol** | **Larnaca** | **Paphos** | **Ammochostos** | **p-value** | **Total** | **Urban** | **Rural** | **p-value** |
| **Vaccines are unnecessary, as viruses can be treated with antibiotics.** | | | | | | | | | | | |
| **T** | 18 (2.6) | 6 (1.7) | 3 (1.6) | 5 (4.7) | 2 (6.5) | 2 (8.7) | 0.30 | 18 (2.7) | 14 (2.6) | 4 (3.1) | 0.53 |
| **F** | 627 (89.6) | 321 (90.2) | 167 (90.8) | 92 (86.8) | 27 (87.1) | 20 (87.0) |  | 598 (89.5) | 481 (89.1) | 117 (91.4) |  |
| **I** | 55 (7.9) | 29 (8.2) | 14 (7.6) | 9 (8.5) | 2 (6.5) | 1 (4.3) |  | 52 (7.8) | 45 (8.3) | 7 (5.5) |  |
| **The effectiveness of vaccines has been demonstrated by epidemiological studies.** | | | | | | | | | | | |
| **T** | 586 (83.8) | 307 (86.2) | 157 (85.3) | 78 (74.3) | 26 (83.9) | 18 (78.3) | **<0.01** | 556 (83.4) | 456 (84.6) | 100 (78.1) | 0.20 |
| **F** | 17 (2.4) | 3 (0.8) | 7 (3.8) | 5 (4.8) | 1 (3.2) | 1 (4.3) |  | 18 (2.7) | 13 (2.4) | 5 (3.9) |  |
| **I** | 23 (3.3) | 8 (2.3) | 4 (2.2) | 5 (5.7) | 4 (12.9) | 1 (4.3) |  | 93 (13.9) | 70 (13.0) | 23 (18.0) |  |
| **Systematic vaccination helped to reduce or eliminate many infectious diseases worldwide.** | | | | | | | | | | | |
| **T** | 654 (93.6) | 340 (95.8) | 174 (94.6) | 92 (86.8) | 27 (87.1) | 21 (91.3) | **<0.01** | 622 (93.3) | 508 (94.3) | 114 (89.1) | **<0.01** |
| **F** | 22 (3.2) | 7 (2.0) | 6 (3.3) | 8 (7.6) | 0 (0.0) | 1 (4.3) |  | 22 (3.3) | 18 (3.3) | 4 (3.1) |  |
| **I** | 23 (3.3) | 8 (2.3) | 4 (2.2) | 5 (5.7) | 4 (12.9) | 1 (4.3) |  | 23 (3.5) | 13 (2.4) | 10 (7.8) |  |
| **Vaccination can be done in summer.** | | | | | | | | | | | |
| **T** | 536 (76.7) | 269 (75.6) | 149 (81.0) | 75 (71.4) | 25 (80.7) | 18 (78.3) | 0.45 | 512 (76.8) | 415 (77.0) | 97 (75.8) | 0.96 |
| **F** | 20 (2.9) | 11 (3.1) | 3 (1.6) | 6 (5.7) | 0 (0.0) | 0 (0.0) |  | 20 (3.0) | 16 (3.0) | 4 (3.1) |  |
| **I** | 143 (20.5) | 76 (21.3) | 32 (17.4) | 24 (22.9) | 6 (19.4) | 5 (21.7) |  | 135 (20.2) | 108 (20.0) | 27 (21.1) |  |
| **Vaccination can be done when my child has a cold.** | | | | | | | | | | | |
| **T** | 113 (16.1) | 54 (15.2) | 37 (20.1) | 17 (16.0) | 3 (9.7) | 2 (8.7) | 0.26 | 108 (16.2) | 92 (17.0) | 16 (12.5) | 0.24 |
| **F** | 510 (72.9) | 259 (72.8) | 124 (67.4) | 83 (78.3) | 24 (77.4) | 20 (87.0) |  | 487 (72.9) | 386 (71.5) | 101 (78.9) |  |
| **I** | 77 (11.0) | 43 (12.1) | 23 (12.5) | 6 (5.7) | 4 (12.9) | 1 (4.4) |  | 73 (10.9) | 62 (11.5) | 11 (8.6) |  |
| **Vaccination can be done when my child has a fever (>38°C).** | | | | | | | | | | | |
| **T** | 19 (2.7) | 10 (2.8) | 6 (3.3) | 1 (0.9) | 0 (0.0) | 2 (9.1) | 0.33 | 18 (2.7) | 14 (2.6) | 4 (3.2) | 0.57 |
| **F** | 632 (90.4) | 326 (91.6) | 161 (87.5) | 99 (93.4) | 28 (90.3) | 18 (81.8) |  | 604 (90.6) | 487 (90.2) | 117 (92.1) |  |
| **I** | 48 (6.9) | 20 (5.6) | 17 (9.2) | 6 (5.7) | 3 (9.7) | 2 (9.1) |  | 45 (6.8) | 39 (7.2) | 6 (4.7) |  |
| **Vaccine for measles/ rubella/ rubella/ mumps (MMR) is associated with autism.** | | | | | | | | | | | |
| **T** | 38 (5.4) | 11 (3.1) | 13 (7.1) | 8 (7.6) | 5 (16.1) | 1 (4.3) | **<0.01** | 38 (5.7) | 30 (5.6) | 8 (6.3) | 0.26 |
| **F** | 366 (39.1) | 208 (58.8) | 91 (49.5) | 43 (40.6) | 15 (48.4) | 9 (39.1) |  | 350 (52.6) | 291 (54.1) | 59 (46.1) |  |
| **I** | 294 (42.1) | 135 (38.1) | 80 (43.5) | 55 (51.9) | 11 (35.5) | 13 (56.5) |  | 278 (41.7) | 217 (40.3) | 61 (47.7) |  |
| **Children would be more resistant if they were not vaccinated.** | | | | | | | | | | | |
| **T** | 36 (5.2) | 15 (4.2) | 8 (4.4) | 11 (10.5) | 1 (3.2) | 1 (4.4) | **<0.01** | 35 (5.3) | 28 (5.2) | 7 (5.5) | 0.93 |
| **F** | 582 (83.3) | 306 (86.0) | 142 (77.2) | 89 (84.8) | 25 (80.7) | 20 (87.0) |  | 554 (83.1) | 450 (83.3) | 104 (81.9) |  |
| **I** | 81 (11.6) | 35 (9.8) | 34 (18.5) | 5 (4.8) | 5 (16.1) | 2 (8.7) |  | 78 (11.7) | 62 (11.5) | 16 (12.6) |  |
| **Many vaccines are given too early, leaving the children's immune system, unable to develop.** | | | | | | | | | | | |
| **T** | 44 (6.3) | 13 (3.7) | 15 (8.2) | 11 (10.4) | 3 (9.7) | 2 (8.7) | 0.15 | 44 (6.6) | 33 (6.1) | 11 (8.6) | 0.57 |
| **F** | 475 (68.0) | 253 (71.3) | 116 (63.0) | 68 (64.2) | 23 (74.2) | 15 (65.2) |  | 451 (67.6) | 365 (67.7) | 86 (67.2) |  |
| **I** | 180 (25.8) | 89 (25.1) | 53 (28.8) | 27 (25.5) | 5 (16.1) | 6 (26.1) |  | 172 (25.8) | 141 (26.2) | 31 (24.2) |  |
| **The doses of chemicals that are used in the vaccines are dangerous for humans.** | | | | | | | | | | | |
| **T** | 40 (5.7) | 11 (3.1) | 12 (6.5) | 10 (9.5) | 4 (12.9) | 3 (13.0) | **<0.01** | 40 (6.0) | 29 (5.4) | 11 (8.6) | 0.15 |
| **F** | 445 (63.7) | 247 (69.4) | 114 (62.0) | 54 (51.4) | 16 (51.6) | 14 (60.9) |  | 426 (63.8) | 353 (65.4) | 73 (57.0) |  |
| **I** | 214 (30.6) | 98 (27.5) | 58 (31.5) | 41 (39.1) | 11 (35.5) | 6 (26.1) |  | 202 (30.2) | 158 (29.3) | 44 (34.4) |  |
| **Vaccination increases the appearance of allergies.** | | | | | | | | | | | |
| **T** | 40 (5.7) | 14 (3.9) | 12 (6.5) | 10 (9.4) | 4 (12.9) | 0 (0.0) | 0.23 | 41 (6.2) | 30 (5.6) | 11 (8.7) | 0.34 |
| **F** | 349 (49.9) | 187 (52.5) | 89 (48.4) | 49 (46.2) | 14 (45.2) | 10 (45.5) |  | 331 (49.6) | 273 (50.6) | 58 (45.7) |  |
| **I** | 310 (44.3) | 155 (43.5) | 83 (45.1) | 47 (44.3) | 13 (41.9) | 12 (54.6) |  | 295 (44.2) | 237 (43.9) | 58 (45.7) |  |
| **There is a vaccine to prevent cervical cancer.** | | | | | | | | | | | |
| **T** | 682 (95.6) | 351 (98.6) | 177 (96.2) | 102 (97.1) | 29 (93.6) | 23 (100.0) | 0.34 | 653 (97.9) | 527 (97.6) | 126 (99.2) | 0.49 |
| **F** | 3 (0.4) | 0 (0.0) | 2 (1.1) | 1 (1.0) | 0 (0.0) | 0 (0.0) |  | 3 (0.5) | 3 (0.6) | 0 (0.0) |  |
| **I** | 14 (2.0) | 5 (1.4) | 5 (2.7) | 2 (1.9) | 2 (6.5) | 0 (0.0) |  | 11 (1.7) | 10 (1.9) | 1 (0.8) |  |
| **Vaccination is not needed for diseases that have disappeared.** | | | | | | | | | | | |
| **T** | 52 (7.4) | 23 (6.5) | 13 (7.1) | 10 (9.4) | 5 (16.1) | 1 (4.3) | 0.12 | 52 (7.8) | 42 (7.8) | 10 (7.8) | 0.65 |
| **F** | 519 (74.1) | 276 (77.5) | 137 (74.5) | 69 (65.1) | 22 (71.0) | 15 (65.2) |  | 494 (74.0) | 403 (74.6) | 91 (71.1) |  |
| **I** | 129 (18.4) | 57 (16.0) | 34 (18.5) | 27 (25.5) | 4 (12.9) | 7 (30.4) |  | 122 (18.3) | 95 (17.6) | 27 (21.1) |  |
| Abbreviations: T, true; F, false; I, I don’t know; Bold font indicates statistical significance (p<0.05). | | | | | | | | | | | |
